# Case Report: A spinal infection with bilateral psoas abscesses was treated with NPWT to enhance the local infection by increasing the infiltration of neutrophil cells and draining the pus

**DOI:** 10.3389/fcimb.2023.1228376

**Published:** 2023-08-04

**Authors:** Jianhua Li, Zhengqi Chang

**Affiliations:** Department of Orthopedics, 960th Hospital of PLA, Jinan, China

**Keywords:** neutrophil, spinal infections, bilateral psoas abscess, lateral approach, negative pressure wound therapy (NPWT)

## Abstract

Treatment of spinal brucellosis with bilateral psoas abscess is a challenging clinical endeavor. We retrospectively evaluated a case of lumbar infection and bilateral psoas abscess, and was effectively managed through a unilateral extreme lateral approach with the aid of NPWT for bilateral drainage. We hypothesize that NPWT can influence the Piezo1 receptor of neutrophils and further influence the interaction between neutrophils and endothelial cells to promote the clearance of infected lesions, and this phenomenon is also observed in pathological slides. This proves that NPWT can rapidly enhance the recruitment of neutrophils in the infected area and improve the local immune response, and after a year of reassessment and tracking, Bilateral drainage using NPWT via a unilateral Extreme Lateral Approach could acquire satisfactory surgical outcomes, can be used as a treatment modality for lumbar infection with bilateral psoas abscesses.

## Introduction

Diagnosing spinal infections can be a challenge as they typically start off with subtle and gradual symptoms, thus necessitating a heightened level of vigilance. Brucellar spondylitis is a type of spinal infection that can result in the development of psoas abscesses. Literature accounts indicate that abscesses are present in 7.1% of brucellosis patients (Bosilkovski et al., 2004). A study of 124 patients with psoas abscesses revealed that Brucella was the underlying cause in 5.7% of the cases, and the source of the infection was attributed to the skeletal system in each instance (Lopez et al., 2009). According to a study conducted by Harrigan RA and colleagues, the mortality rate of secondary psoas abscess was found to be 18.9% (Harrigan et al., 1995).

Many patients with spinal infection can be treated with non-invasive drug therapy (Babic and Simpfendorfer, 2017; Bhattacharyya and Bradshaw, 2021; Sun et al., 2023). However, the restricted blood supply and the enclosed anatomy of the intervertebral disc tissue often result in a low concentration of the medication in the lesion, making conservative treatment often ineffective. If the patient is experiencing clear signs of pain, vertebral instability, abscess formation, and neurological symptoms during the course of conservative treatment, it may be necessary to switch to a surgical approach (Zhao et al., 2020). Traditional open surgery is capable of eliminating the infected lesion, however it can also cause considerable injury to the patient. The intricate anatomy of the spine and the fluidity of the abscess make it impossible for a single-stage bone grafting, fusion, and internal fixation to fully eradicate the infected lesion of the spine, thus leaving a potential source of the disease’s recurrence. Due to the patient’s prolonged spinal infection and bilateral psoas major abscess, her overall physical health and nutritional status had deteriorated, making it impossible for him to endure a single-stage bone graft fusion and internal fixation procedure that was both lengthy and traumatic. Minimally invasive surgery, while causing minimal trauma, is unable to completely remove the infection lesions that have spread into the intervertebral space and both psoas muscles. To solve this problem, we innovatively formulated a bilateral drainage scheme using NPWT via a unilateral Extreme Lateral Approach that was successful and yielded a desirable result. In addition, we found that NPWT can significantly enhance neutrophil recruitment during treatment, and neutrophils are the most important innate immune cells in the body defense against invading pathogenic microorganisms and play an essential role in the early defense against tissue injury by their adhesion, exudation, and activation to apoptosis. Therefore, we analyzed and reasoned that NPWT has a good effect of enhancing local immune response and curing spinal infection. It is reported as follows.

## Case presentations

**Figure 1:**
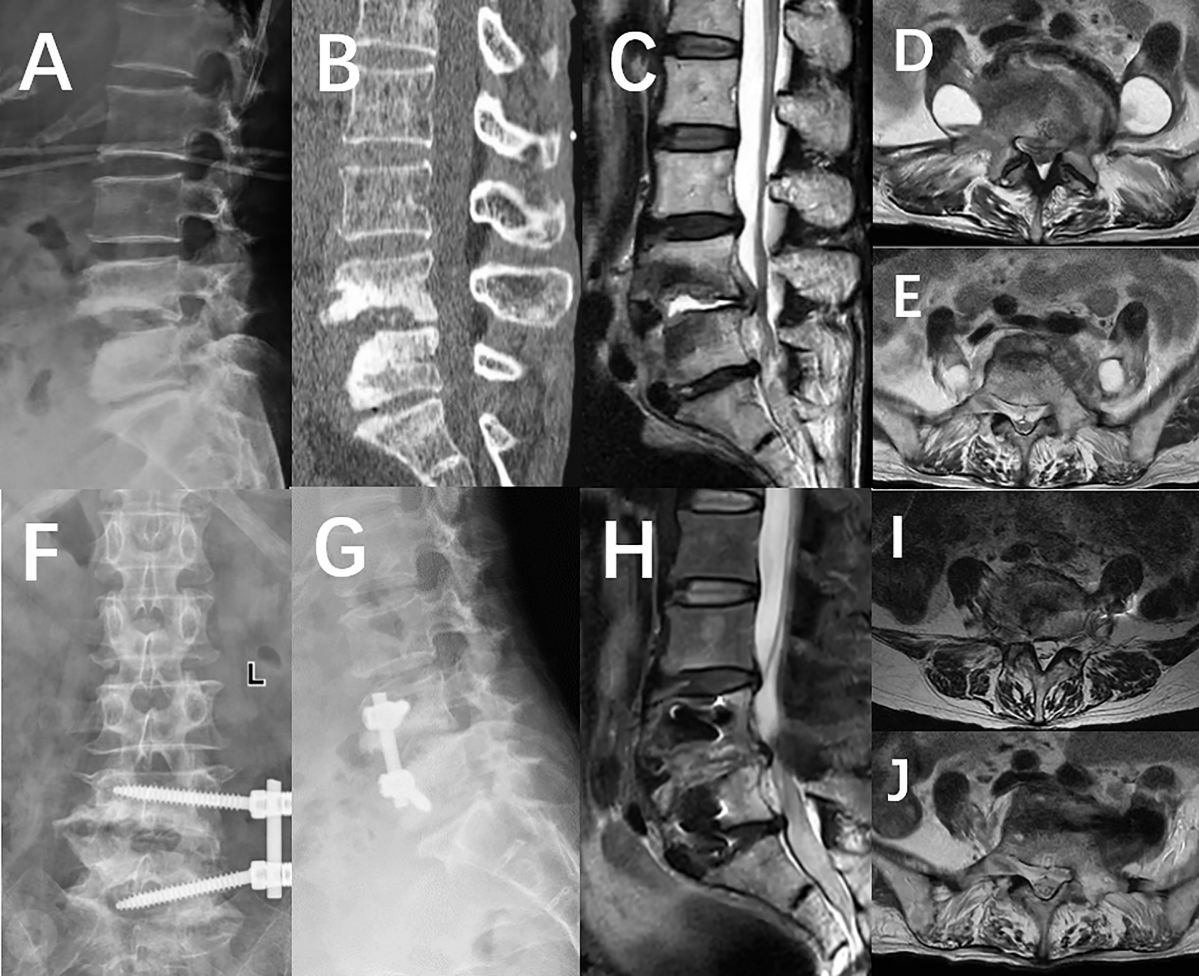
**(A)** X-ray imaging demonstrated destruction of the L4 and L5 vertebral bodies, as well as a decrease in the intervertebral space height. **(B)** The CT scan revealed a significant amount of bone erosion in the L4 and L5 vertebrae, resembling the effects of a worm-eaten surface. **(C)** Magnetic Resonance Imaging demonstrated the formation of an abscess in the L4/5 intervertebral space, in the spinal canal, and around the vertebral body. **(D, E)** Abscesses accumulated in the psoas bilaterally and spread caudally along the intermuscular space. **(F, G)** An X-ray taken a week after the operation revealed that the internal fixation was in the correct position. **(H–J)** The postoperative MRI indicated that the abscess had been completely drained and cleared after the Negative Pressure Wound Therapy treatment.

A 64-year-old woman with a history of contact with livestock such as sheep and cattle presented to our department with complaints of low back pain and accompanying pain and numbness in both lower limbs for a period of three months, with an increase in severity over the past month. Physical examination revealed that the patient had neurogenic claudication as well as lower back existed pressure and percussion pain radiating to the lower limbs and reaching the ankle joint bilaterally; decreased bilateral extensor hallucis longus muscle power (grade 3); Fabere test was positive bilaterally; Straight leg raising was restricted to <70° bilaterally, the Babinski sign was negative bilaterally. Upon admission, the patient’s BMI was 18, hemoglobin was 98 g/L, albumin was 31.4 g/L, ESR was 49 mm/h, and CRP was 87.28 mg/L. The Visual Analogue Scale (VAS) score was 8, and the American Spinal Injury Association grade (ASIA) was classified as D. X-ray imaging of the lumbar spine demonstrated a narrowing in the intervertebral space and destruction of the L4 and L5 vertebral bodies. CT of the lumbar spine revealed evidence of a “worm-eaten” destruction to the L4 and L5 vertebral bodies, with marginal sclerotic bone formation and a reduction in intervertebral space height. MRI revealed the presence of an abscess in the lumbar spine, located around the vertebral body, inside the spinal canal, and in the bilateral psoas muscles (**Figures 1A–E**). The Brucella agglutination test showed a positive outcome. The patient has been diagnosed with lumbar Brucellosis infection combined with bilateral psoas abscesses. After admission, a fourteen-day course of treatment is advised which includes doxycycline (100 mg, oral, bid, 3 months) in combination with gentamicin (5 mg/kg, intramuscular injection, Qd, 1 week) and rifampicin (10 mg/kg, up to 900 mg, oral, 3 months). Because considering old age, underlying disease, complexity of the abscess chamber, and Severity of vertebral damage, we decided to performed a drainage using NPWT via a unilateral extreme lateral approach to manage the spinal infection with bilateral psoas abscess.

## Technical note

**Figure 2:**
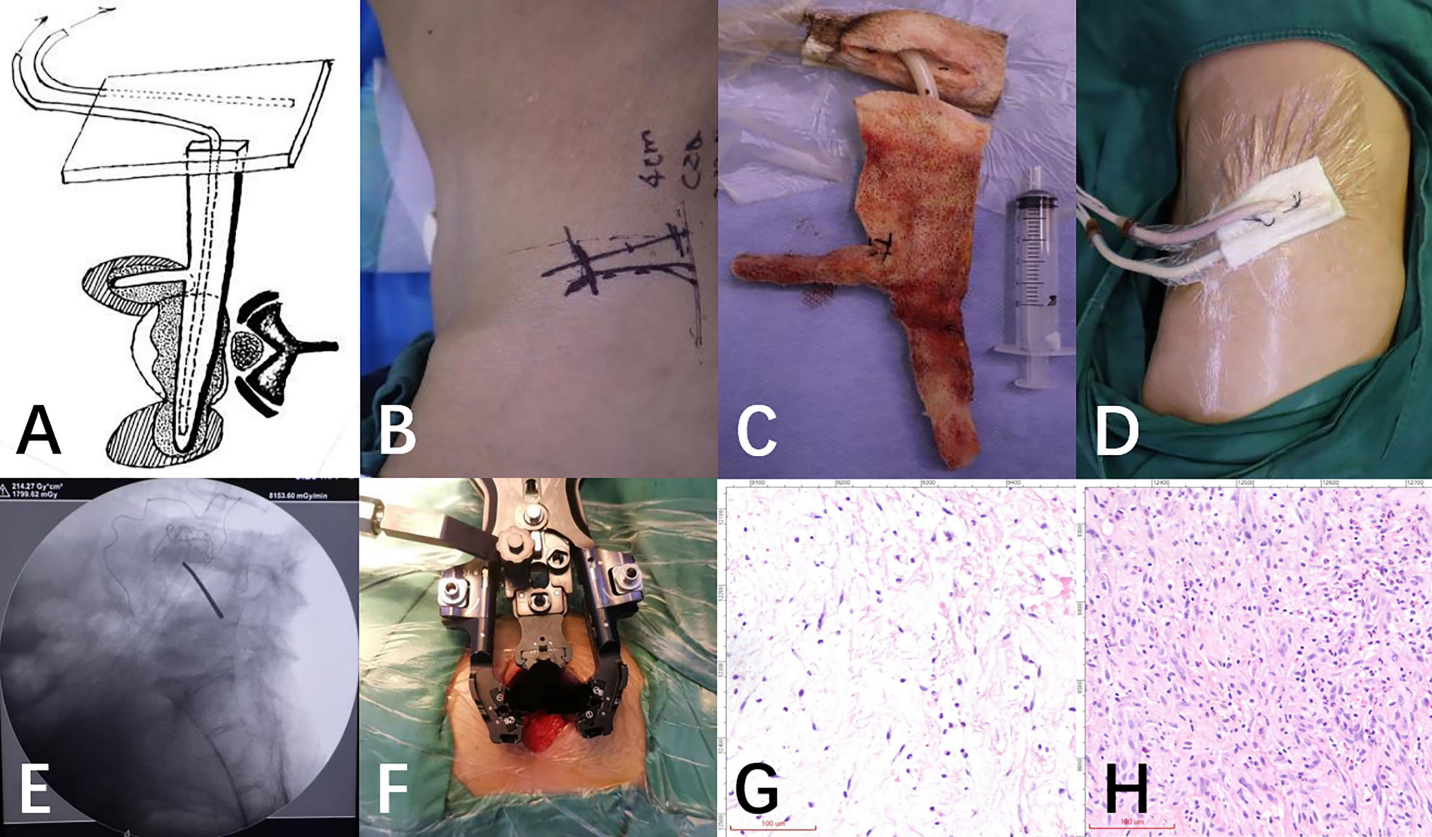
**(A)** The following schematic diagram demonstrates the placement of VSD sponge strips. **(B)** Right lateral position, an extreme lateral surgical incision on the upper border of the anterior superior iliac crest can be designed. **(C)** The VSD sponge strips were removed from the gap and bilateral psoas abscess prior to the bone graft fusion and internal fixation. **(D)** The photos taken after the installation of the VSD negative pressure device showed the successful implementation. **(E)** C-arm fluoroscopy verified the L4/5 intervertebral space. **(F)** To increase the surgical field, dilation channels were placed in a series of steps. **(G, H)** Changes of neutrophils before and after NPWT treatment.

Under general anesthesia, patients were placed in a right lateral position with her waist raised. Utilizing fluoroscopy, the L4/5 space was pinpointed and indicated on the body’s surface. Sanitize the operative area and lay the drape, then cut a 4-centimeter-long incision at the designated waist area. Cut the skin and underlying tissue in order, and then carefully remove the tissue behind the peritoneum to expose the psoas muscle. Then, follow the anterior 1/3 of psoas muscle and gently dissect to uncover the capsule of a psoas abscess. An incision was made in the abscess capsule, allowing for the extraction of the focal abscess. The vertebral body and the L4/5 intervertebral space were then revealed, and an automatic distractor was installed and secured. Sever the fibrous ring and eradicate all the inflamed and necrotic tissue inside. Following the penetration of the contralateral fibrous ring, the contralateral psoas abscess should be accessed and irrigated with a copious amount of Dodecyl dimethyl benzyl ammonium bromide and normal saline to flush the intervertebral space. The preoperatively designed VSD sponge should be placed into the bilateral psoas major and L4/5 intervertebral space, as illustrated in **Figure 2**, to guarantee efficient drainage of the intervertebral space and bilateral psoas muscle abscesses (**Figures 2A–F**). The body is covered with another VSD sponge, and the negative pressure is connected upon the sealing of the film. Ensure that the drainage is flowing smoothly, the VSD device is properly sealed, and there are no air leaks, then the operation is complete. During the operation, approximately 80ml blood was lost without any blood transfusion.

After the initial VSD device has been in place for a week, it should be replaced with a new one. Two weeks after the initial operation, the patient underwent a bone graft fusion and internal fixation. Under general anesthesia, the iliac crest will be accessed through the original incision and then a lateral screw internal fixation will be implanted in the same position as the preceding operation. The iliac spine was revealed from the prior incision and a piece of self-derived iliac bone of the appropriate dimensions, along with a sufficient amount of spongy bone, was removed using a bone knife as a reserve. After the automatic distraction device was put in place, the L4/5 interval was revealed again, and autologous iliac bone block and cancellous bone were inserted into the opening. Secure two screws in the L4 and L5 positions, and attach titanium rods of the correct length to complete the lateral internal fixation.

## Postoperative course and follow-up

Histological analysis of tissue samples taken before and after each surgery demonstrate a significant increase (70% to 89%) in the presence of neutrophils (**Figures 2G, H**). Upon discharge, the patient was no longer suffering from low back pain, and was able to move around and walk without any help, and the VAS score had dropped from 8 to 1, with the ASIA classification being E. The X-ray film and MRI of the lumbar spine revealed that the abscess had vanished and the fixation was satisfactory (**Figures 1F–J**).

**Figure 3:**
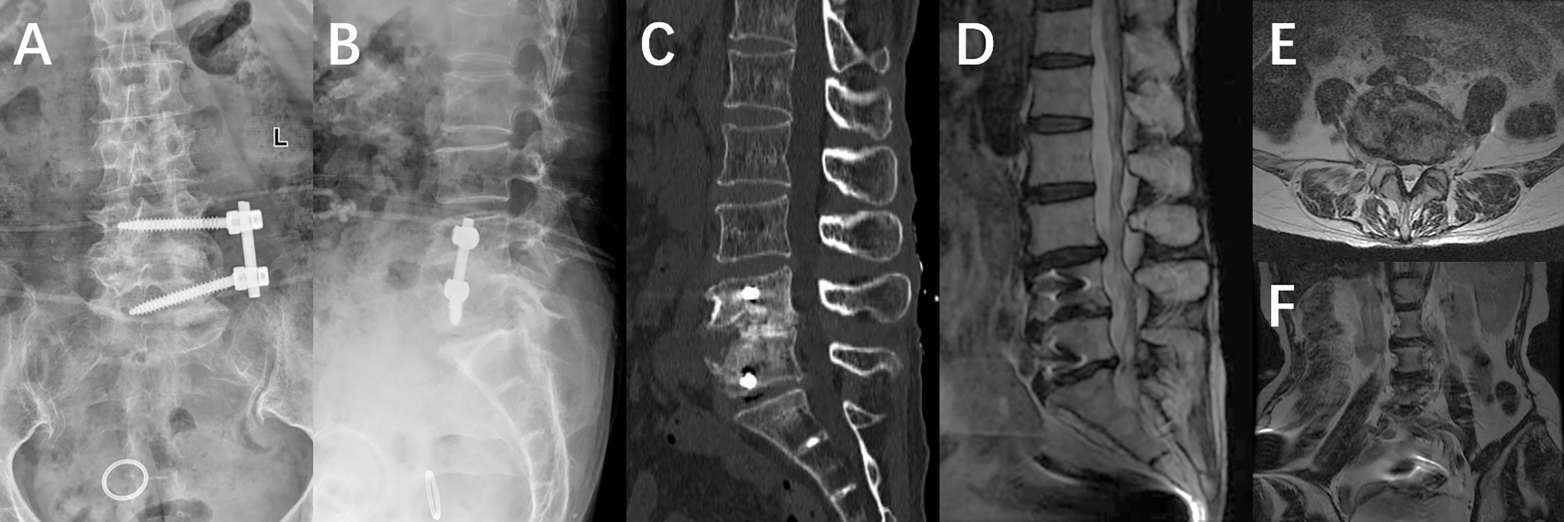
**(A, B)** An X-ray taken a week after the operation revealed that the internal fixation was in the correct position. **(C–F)** The postoperative MRI indicated that the abscess had been completely drained and cleared after the Negative Pressure Wound Therapy treatment.

One year after the operation, the patient’s BMI index was 24.91, hemoglobin level was 128g/L, albumin level was 40.7g/L, ESR was 11mm/h, and CRP was 6mg/L. Re-examination of X-ray, CT, and MRI scans revealed that the internal fixation position was satisfactory and the bone graft had fused. In addition, abnormal signals in the lumbar spine, spinal canal, and psoas major muscle had disappeared (**Figure 3**).

## Discussion

Spinal infection accompanied by psoas abscess is a surgical emergency due to threatening life. Compared to unilateral psoas abscess, bilateral psoas abscess is less common. Principles of managing spinal infection is to achieve spinal stability while controlling the infection (Duarte and Vaccaro, 2013). Surgery at an early stage can help to prevent the further propagation of abscess ulceration, which can lead to sepsis, as well as permanent nerve damage due to spinal cord compression.

Deng L et al. (Deng et al., 2020) employed XLIF in conjunction with percutaneous pedicle screw technology to conduct tuberculosis debridement, discectomy, and intervertebral fusion under direct vision, which succeeded in curing a patient suffering from thoracic tuberculosis. Yangbin Chen et al. (Chen et al., 2017) suggested that lumbar brucella spondylitis could be treated with a one-time debridement, autogenous bone grafting, and instrumentation through a posterior approach. Li J et al. (Li et al., 2014) successfully treated thoracolumbar tuberculosis with bilateral lumbar muscle abscess through a single-stage posterior approach that enabled spinal cord decompression, spinal fusion, and spinal stabilization, while also restoring spinal alignment. Koichi Kodama et al. (Kodama et al., 2014) treated A 67-year-old man presented with bilateral psoas abscesses secondary to L1–L2 pyogenic discitis by performed surgical drainage of bilateral psoas abscesses by retroperitoneoscopy.

To the best of our knowledge, there is no published material on combining XLIF and NPWT for staged treatment of spinal infection with bilateral psoas major infection. (**Supplementary Table 1**) Nevertheless, our innovative unilateral approach combined with bilateral drainage was triumphant in curing the infected lesion. The abscess was substantial, making the first phase of bilateral psoas debridement + bone graft fusion and internal fixation challenging. The field of view was limited due to the minimally invasive endoscopic technique, and the cavities of the lesion were intricate, making it impossible to completely remove the lesion with minimally invasive endoscopic surgery. The imaging report reveals that the patient’s L4 and L5 vertebrae have experienced bone destruction that appears to be “worm-eaten,” and the height of the intervertebral space has been reduced. Visible signs of an abscess can be observed in the L4/5 intervertebral space, the spinal canal and the bilateral psoas muscles. Taking into account the particular characteristics of the location of the complex and multi-cavity abscess lesions, we innovated a design for the shape of the negative pressure sponge that is necessary for their drainage.

The Extreme Lateral Interbody Fusion approach offers a less invasive alternative to the traditional anterior, posterior, or combined anterior-posterior approaches. This technique also causes minimal disruption to the stability of the spine, thus avoiding many complications associated with traditional open surgery (Ozgur et al., 2006; Deng et al., 2020). NPWT is a beneficial technique for the removal of necrotic tissue fragments from a wound, as well as the efficient drainage of wound exudate, which can expedite wound healing (Duan et al., 2020). Evidence suggests that NPWT is a successful treatment for bone infection, with wound healing and infection healing times drastically reduced in comparison to traditional dressings (Webb, 2017); Dates from reports demonstrates that the use of Negative Pressure Wound Therapy to treat spinal infection in the infected intervertebral space and abscess is both safe and effective (Wang et al., 2022; Xing et al., 2022). By combining XLIF and NPWT, the shortcomings of traditional open surgery and endoscopic minimally invasive surgery can be effectively addressed in patients with spinal infection complicated complex conditions.

In the process of infection or tissue injury, neutrophils are the first to reach the sites of inflammation. Neutrophils phagocytose pathogenic microorganisms by recognizing specific signals such as chemokines or bacterial products released near the lesion. In addition, endothelial migration is a key physiological process in angiogenesis, growth, development, and a variety of disease processes. By attaching and passing through the endothelial lining at the base of the blood vessel, mass of neutrophils migrate and infiltrate the area of inflammation.

The interaction between neutrophils and endothelial cells may help enhance the immune response. However, the molecular mechanism of this interaction has not been completely elucidated and therefore more novel studies are needed to understand the underlying mechanisms of neutrophil-endothelial cell interactions, which will provide new strategies for the diagnosis and prognosis of infectious diseases. We believe that the Piezo1 may contribute to this immune process.

Piezo1, a receptor for the mechanical forces of the outside world, facilitates the flow of Ca2+ from outside the cell to the cells, enabling the conversion of the mechanical forces of the external environment into the biological signals inside (Solis et al., 2019). Mechanical Forces, including Hydrostatic Pressure, Laminar Flow Forces and Cellular Stretch, activate the Piezo1 channel, induce immune cells to exhibit different immune responses (Ma et al., 2021) and regulate the clearance of pathogens by immune cells (Geng et al., 2021). Under the influence of NPWT, the cytoskeletal-extracellular matrix interactions promote the mass recruitment of neutrophils by enhancing intracellular signals, activating surface receptors, triggering migration, and cell-to-cell communication (Orsini et al., 2021). We also found this phenomenon in the pathological sections of this patient (**Figures 2G, H**), which further demonstrated that NPWT has the advantage of promoting neutrophil recruitment and enhancing local immune response.

## Conclusion

We performed a staged surgery using a unilateral extreme lateral approach with the aid of NPWT for bilateral drainage for spinal infection with bilateral psoas abscess, NPWT has the potential to bolster the local immune response with a particular focus on neutrophil infiltration. This method can be an option for traditional anterior, posterior, or combined anterior-posterior approaches, but the surgical indications of the patients need to be selected strictly.

## Data availability statement

The raw data supporting the conclusions of this article will be made available by the authors, without undue reservation.

## Ethics statement

The patient in this case report gave consent for her data to be included in this report. Written informed consent for publication of her clinical details and/or clinical images was obtained from the patient/parent/guardian/relative of the patient.

## Author contributions

JL and ZC made substantial contributions to acquisition, analysis and interpretation of data. ZC was responsible for the conception and design of the study and the drafting and writing of this manuscript. JL was a surgical assistant. All authors contributed to the article and approved the submitted version.
