# Supplementary material for: Case Report: A spinal infection with bilateral psoas abscesses was treated with NPWT to enhance the local infection by increasing the infiltration of neutrophil cells and draining the pus
Source: Front Cell Infect Microbiol. 2023 Aug 4;13:1228376. doi: 10.3389/fcimb.2023.1228376 (PMC10436603; doi:10.3389/fcimb.2023.1228376)
Supplement: Supplementary file 1 [file Table_1.docx]

| Sequence | author | year | Diseases | operation | Number of  cases | mean age（year） | VAS | |  | ASIA | |  | Cobb | | Total operation  time (min) | Total  bleeding  (mL) | hospital stays  (day) | Antibiotic application time (month) | Bone healing time (month) | Follow-up time  (month) | Complication |
| --- | --- | --- | --- | --- | --- | --- | --- | --- | --- | --- | --- | --- | --- | --- | --- | --- | --- | --- | --- | --- | --- |
|  |  |  |  |  |  |  | pre-operation | The last |  | pre-operation | The last |  | pre-operation | The last |  |  |  |  |  |  |  |
| 1 | Yangbin Chen^[7]^ | 2017 | lumbar brucella spondylitis | One-stage  posterior debridement, autogenous bone graft and instrumentation | 24 | 56.1 ± 10.7 | 7.5 ± 1.4 | 0.8 ±0.7 |  | C6 D10 E8 | D2 E22 |  | 18.4 ± 4.6° | 21.1 ± 3.7° | 229.6 ± 56.5 | 430.2 ± 171.9 | 16.0 ± 3.4 | 6.5 ± 2.5 | 6.8 ± 1.6 | 14.3 ± 3.5 | NO |
| 2 | Rugang Zhao^[1]^ | 2020 | Lumbar Brucella Spondylitis | one-stage posterior debridement, instrumentation, and interbody fusion with PEEK cages | 61 | 56.33 ± 9.16 | 5.85 ± 1.26 | 1.69 ± 1.35 |  | C 10 D13 E38 | D4 E57 |  | — | — | 124.10 ± 34.52 | 346.23 ± 153.89 | — | 7 | — | 14.45 ± 4.25 | Local infection 1 |
| 3 | Xin Hua Yin^[8]^ | 2018 | lumber brucella spondylitis | one-stage anterior internal fixation, debridement, and bone fusion | 16 | 45.0±10.3 | 7.1±2.9 | 0.8±3.4 |  | B1 C4 D6 E5 | D2 E14 |  | 20.7±9.8° | 8.1±1.3° | 237.4±29.5 | 580.2±140.3 | — | 1.5 | 4.8±1.3 | 35.3±8.1 | Wound infection 1 Pain of graft harvesting site 1 |
| 4 | Yakefu Abulizi^[9]^ | 2017 | lumbosacral spinal brucellosis | single-stage transforaminal decompression, debridement, interbody fusion, and posterior instrumentation | 32 | 53.7 ± 8.7 | 5.19 ± 1.47 | 0.47 ± 0.67 |  | — | — |  | — | — | 133.1 ± 36.6 | 378.1 ± 187.9 | 11.5 ± 1.9 | 3 | — | 24.9 ± 8.2 | Wound infection 2 Sinus formation 1 |
| 5 | Peng Na^[10]^ | 2020 | Lumbar brucella spondylitis | one-stage anterior debridement, fusion, and fixation | 13 | 39.8±12.2 | 7.1±1.2 | 1.2±0.8 |  | — | — |  | 14.6±1.2 | 7.7±1.5 | 234±36.2 | 430.0±75.1 | 13.4±1.6 | 3-6 | 7.9±1.9 | 31.6±6.3 | Wound infection 1 Loosening of fixation 1 |
|  |  |  | Lumbar brucella spondylitis | posterior debridement, bone graft, and fixation | 14 | 43.5±11.3 | 6.9±0.9 | 1.1±0.9 |  | — | — |  | 15.4±1.8 | 6.6±0.9 | 206.7±26.3 | 350±70.7 | 14.7±1.7 | 3-6 | 8.8±1.4 | 32.8±4.8 | NO |

# Table 1 The literature reports on the specific conditions of surgery for patients with lumbar brucella spondylitis in the past 10 years, including operating time, blood loss, and complications.
